# Preservation of the Photoreceptor Inner/Outer Segment Junction in Dry Age-Related Macular Degeneration Treated by Rheohemapheresis

**DOI:** 10.1155/2015/359747

**Published:** 2015-08-17

**Authors:** Eva Rencová, Milan Bláha, Jan Studnička, Vladimír Bláha, Miriam Lánská, Ondřej Renc, Alexander Stepanov, Věra Kratochvílová, Hana Langrová

**Affiliations:** ^1^Department of Ophthalmology, Lékařská Fakulta, Fakultní Nemocnice, Sokolská 581, 500 09 Hradec Králové, Czech Republic; ^2^Fourth Department of Internal Medicine–Hematology, Lékařská Fakulta, Fakultní Nemocnice, Sokolská 581, 500 09 Hradec Králové, Czech Republic; ^3^Department of Gerontology and Metabolic Care, Lékařská Fakulta, Fakultní Nemocnice, Sokolská 581, 500 09 Hradec Králové, Czech Republic; ^4^Department of Radiology, Lékařská Fakulta, Fakultní Nemocnice, Sokolská 581, 500 09 Hradec Králové, Czech Republic

## Abstract

*Aim*. To evaluate the long-term effect of rheohemapheresis (RHF) treatment of age-related macular degeneration (AMD) on photoreceptor IS/OS junction status. *Methods*. In our study, we followed 24 patients with dry AMD and drusenoid retinal pigment epithelium detachment (DPED) for a period of more than 2.5 years. Twelve patients (22 eyes) were treated by RHF and 12 controls (18 eyes) were randomized. The treated group underwent 8 RHF standardized procedures. We evaluated best-corrected visual acuity, IS/OS junction status (SD OCT), and macular function (multifocal electroretinography) at baseline and at 2.5-year follow-up. *Results*. RHF caused a decrease of whole-blood viscosity/plasma viscosity at about 15/12%. BCVA of treated patients increased insignificantly (*P* = 0.187) from median 74.0 letters (56.2 to 81.3 letters) to median 79.0 letters (57.3 to 83.4 letters), but it decreased significantly from 74.0 letters (25.2 to 82.6 letters) to 72.5 letters (23.4 to 83.1 letters) in the control group (*P* = 0.041). The mfERG responses in the region of eccentricity between 1.8° and 7° were significantly higher in treated patients (*P* = 0.04). *Conclusions*. RHF contributed to sparing of photoreceptor IS/OS junction integrity in the fovea, which is assumed to be a predictive factor for preservation of visual acuity.

## 1. Introduction

Retinal changes associated with the development of age-related macular degeneration (AMD) have become even more important than once described [1]. Even prompt therapy of the wet form of AMD with antivascular, endothelial, growth factor drugs has been shown to ameliorate vision loss [2]. If diagnosis is established earlier—during the dry form of AMD, there is a better chance that visual acuity can be maintained. However, treatment options are still limited at this stage.

Rheohemapheresis (formally known as rheopheresis) has been investigated over the past decade as a possible method of positively affecting AMD in its dry-form stage. Rheohemapheresis is a method of double plasma filtration performed in order to eliminate high-molecular-weight substances—especially proteins such as fibrinogen, *α*_2_-macroglobulin, immunoglobulin M (IgM), thrombomodulin, and low-density (LDL) cholesterol [3–6]. This method leads to the improvement of rheological parameters (reduction of plasma and whole blood viscosity), as well as the improvement of erythrocyte aggregation and their flexibility [6, 7]. It can also lead to a significant improvement of blood flow in the choroid, which is reduced in patients with AMD. Therefore, visual functions can improve [3, 7–9].

When treating our patients suffering from the dry form of AMD accompanied by reticular drusen or even drusenoid detachment of the retinal pigment epithelium, we noticed positive morphological changes of the retina after rheohemapheresis treatment, including the preservation of the photoreceptor inner/outer segment (IS/OS) junction, as well as stabilisation or even improvement of retinal function: best-corrected visual acuity (BCVA) and electroretinography results. A description of the above changes and their correlation is the subject of this work.

## 2. Methods and Subjects of the Study

At our department, we have seven years of experience in treating AMD using rheohemapheresis. So far, 61 patients with the dry form of AMD, soft drusen, confluent soft drusen, and drusenoid pigment epithelium detachment (DPED) have been treated. We perform long-term monitoring of morphological and functional changes in the retina [12, 13].

The interrelationship of morphological changes in the photoreceptor inner and outer segment (IS/OS) junction retinal layer as well as changes in visual acuity and retinal function (electroretinography) was evaluated in the group of 24 patients (40 eyes) who were long-term followed (for 2.5 years or longer). All patients had the dry form of AMD with the presence of drusenoid pigment epithelium detachment. Twelve of these patients (22 eyes) with an average age of 64.3 years (range: 64–83 years) were treated with RHF. Twelve controls (18 eyes) with an average age of 65.6 years (range: 64–83 years) were randomized. Ophthalmologic inclusion criteria were high-risk, preangiogenic form of AMD with soft drusen, reticular drusen, confluent soft drusen, and DPED—in accordance with the EUREYE Study [14]—and ability to complete the series of 8 rheohemapheresis procedures within 10 weeks. Exclusion criteria were any retinal or choroidal disorders other than AMD, optic nerve disorders, glaucoma, conditions limiting the examination of the fundus, and acute bleeding in the studied eye. General exclusion criteria for rheohemapheresis treatment were the usual exclusion criteria of extracorporeal circulation or therapeutic hemapheresis and the absence of peripheral veins suitable for establishing an extracorporeal circuit.

Rheohemapheresis was performed in the treatment group, as described in detail in our previous work [3, 13]. After plasma separation (blood-cell separator, COBE Spectra or Optia, Terumo, Lakewood, CO, USA), the separated plasma was pumped through the rheofilter (Evaflux, Kawasumi, Tokyo, Japan) to remove high-molecular-weight factors. Using this filter, we repeatedly (8 times over the period of 10 weeks) removed a precisely defined spectrum of high-molecular-weight substances, such as fibrinogen, LDL cholesterol, and *α*_2_-macroglobulin, from the blood of the patient, thus reducing blood viscosity in an attempt to improve the perfusion of the retina and the choroid.

**Figure 1:**
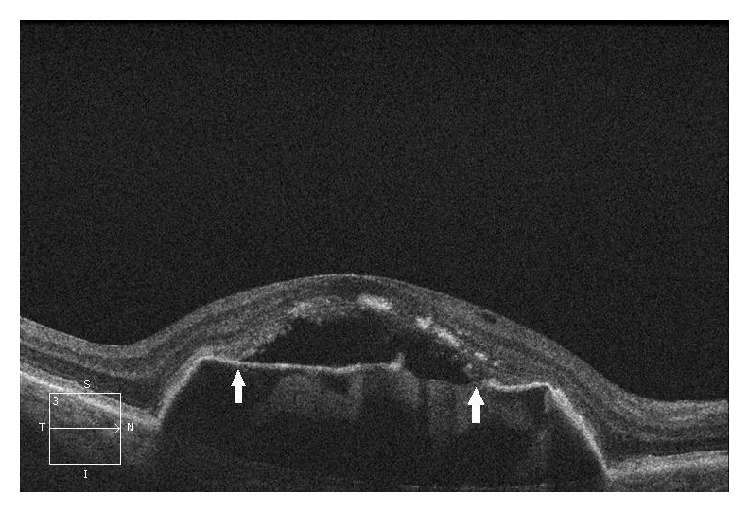
SD-OCT of a patient from the control group—preangiogenic dry from of AMD.SD-OCT of the right eye of a patient from the control group with the dry form of AMD 2.5 years after the start of follow-up, showing persistent DPED. DPED symmetrically transitions into the detachment of the IS/OS photoreceptor junction, localized between the two arrows, where the beginning and end of junction detachment are indicated. In addition, the detached IS/OS photoreceptor junction shows uneven reflectivity and thickness of the retinal layer, a sign of incipient degenerative changes of the IS/OS junction, which are considered to be the result of the lack of junction nutrition due to its increased distance from the RPE [10, 11].

Fundus photography and fluorescein angiography were performed using a digital fundus camera (Zeiss FF 450, Jena, Germany). The DPED area was measured in mm^2^ by fundus photography using VISUPAC software (Zeiss Meditec AG, Jena, Germany), which is acceptable for measuring the area of retinal affections [15]. Spectral domain- (SD-) OCT (Cirrus HD-OCT, Zeiss Meditec, Jena, Germany) with an axial resolution of 6 *μ*m was used to evaluate central retinal thickness. Thickness in the central point of the 1 mm fixation zone was evaluated using 6 radial scans. SD-OCT enabled us to distinguish between DPED and the vascular type of retinal pigment epithelium detachment. A detailed image of the photoreceptor IS/OS junction was obtained using a 5-line raster scan, which precisely shows the shape, coherence, and defects of the photoreceptor IS/OS junction as well as the detachment of this layer from the retinal pigment epithelium (RPE). See Figure 1.

Multifocal ERG (mfERG; RETI-port plus mfERG System, Roland Consult GmbH, Brandenburg, Germany) was performed according to the standards of the International Society for Clinical Electroretinography and Vision (ISCEV) [16, 17]. mfERG traces were recorded from the central 60° of the retina using a resolution of 61-scaled hexagons. We evaluated the amplitudes of the positive peak component from the first-order kernel analysis of the central element, representing the foveal response (0°–1.8°) of the four rings centered on the fovea: 1st ring (1.8°–7.0°), 2nd ring (5°–13°), 3rd ring (11°–22°), and 4th ring (17°–30°). All examinations were performed at baseline and at 2.5-year follow-up.

For statistical analysis, we used nonparametric tests (the Kruskal-Wallis test, Mann-Whitney *U* test, and chi-square approximation). The study protocol was approved by the Institutional Ethics Committee and the reported investigations were in accordance with the principles of the current version of the Helsinki Declaration.

## 3. Results

### 3.1. BCVA

BCVA was evaluated as the number of correctly read ETDRS letters. Median best-corrected visual acuity (BCVA) before rheohemapheresis in the treatment group was 74.0 letters (56.2 to 81.3 letters; 95% CI) and increased to 79.0 letters (57.3 to 83.4 letters; 95% CI), (*P* = 0.187) after 2.5 years. In the control group, mean BCVA decreased from 74.0 letters (25.2 to 82.6 letters; 95% CI) at baseline to 72.5 letters (23.4 to 83.1 letters) after 2.5 years (*P* = 0.041). While the difference in BCVA between the treatment groups was insignificant at baseline (*P* = 0.457), BCVA was significantly higher in the treatment group after 2.5 years (*P* = 0.021).

### 3.2. DPED

At baseline, mean DPED was 3.68 ± 4.45 mm^2^ in treated patients and 4.12 ± 6.64 mm^2^ in controls, reaching the central fovea in all cases. After 2.5 years, the mean DPED area decreased significantly to 0.71 ± 1.27 mm^2^ in the rheohemapheresis group (*P* < 0.001), whereas it increased significantly to 9.19 ± 9.51 mm^2^ in the controls (*P* < 0.01). The differences in size of the DPED area were only insignificant at baseline (*P* = 0.605) and significant after 2.5 years (*P* < 0.001). Reduction of the size of the DPED area after rheohemapheresis was found in 19/22 rheohemapheresis-treated eyes (86.4%), as opposed to only 3/18 eyes (16.7%) in the control group. Enlargement of the DPED area occurred as part of the natural progress of AMD over the same period of 2.5 years in 15/18 (83.3%) eyes in the control group, compared to only 3/22 eyes (13.6%) of treated patients.

### 3.3. IS/OS Receptor Junction

#### 3.3.1. At the Beginning of Follow-Up

The study and control groups were comparable.

- In the group of treated patients, the photoreceptor IS/OS junction was attached to the DPED in 6/22 (27.3%) eyes. (DPED was accompanied by a detachment of the photoreceptor IS/OS junction in the remaining 16/22 eyes, i.e., 72.7%, without defect in 15/22 eyes and with defect in 1 eye).
- In the control group, the photoreceptor IS/OS junction was attached to the DPED in 6/18 (33.3%) (DPED was accompanied by a detachment of the photoreceptor IS/OS junction in the remaining 12/18 eyes (66.6%), without defect in 4 eyes and with defect in 8 eyes).

#### 3.3.2. At the End of Follow-Up

*(1) The Group of Patients.* In the group of treated patients, where the photoreceptor IS/OS junction was intact in 6/22 (27.3%) eyes, it remained without defect even after 2.5 years.

Attachment of the originally detached IS/OS junction occurred in 16 eyes. However, in 7 cases of the attached IS/OS junction, there was a certain residual defect. In one additional eye with the IS/OS junction at baseline, the defect of this layer significantly improved after attachment.

**Scheme 1:**
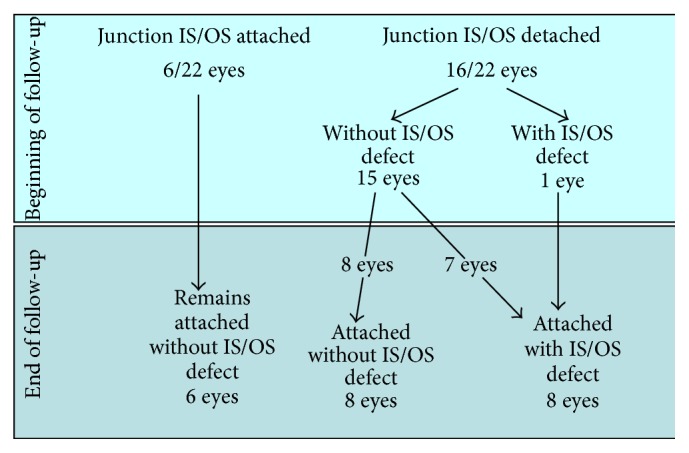
Follow-up results of patients treated with RHF.

*Summary.* Overall, IS/OS junction defects were demonstrated in 8/22 eyes (31.8%). Defects reached the central foveola in 4 eyes (8.8%), thus negatively affecting photoreceptor function and vision. See Scheme 1.

**Scheme 2:**
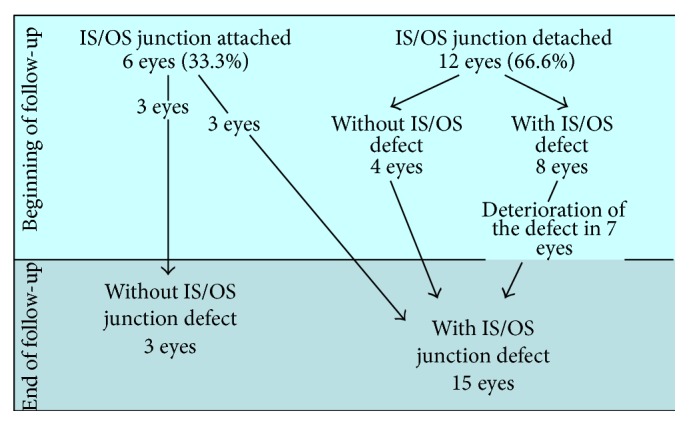
Follow-up results in the control group.

*(2) In the Control Group.* Integrity of the IS/OS junction layer was preserved in only 3/18 eyes (16.7%) after 2.5 years and IS/OS junction defects were diagnosed in 15/18 eyes (83.3%), 12 of which reached the foveolar region and adversely affected vision. See Scheme 2.

*(3) Transition into the Wet Form of AMD.* It is important to note that none of the treated patients progressed into the wet form during the 2.5-year follow-up. In the control group, 6 eyes with detachment of the IS/OS junction at baseline developed choroidal neovascularization (CNV) (confirmed by fluorescein angiography).

### 3.4. Multifocal ERG

The amplitudes of foveal responses and responses in the most peripheral areas of the retina, as shown on the multifocal ERG, changed only slightly during the entire follow-up period in both groups of patients. In the parafoveal and paramacular regions of eccentricity between 1.8° and 13°, we only found an insignificant increase of activity in treated patients. Activity in the same region decreased slightly in the control group. The differences were statistically nonsignificant between groups of patients in both periods of examination, except for the activity in the region of eccentricity between 1.8° and 7°, which was significantly higher in treated patients (*P* = 0.04) at 2.5-year follow-up. The implicit times of the majority of responses increased after 2.5 years in all patients. At baseline, they were significantly longer in the controls (*P* values ranging from < 0.05 to < 0.01), with the exception of the foveal response. At 2.5 years, the foveal response was significantly longer in the control group (*P* = 0.035).

**Figure 2:**
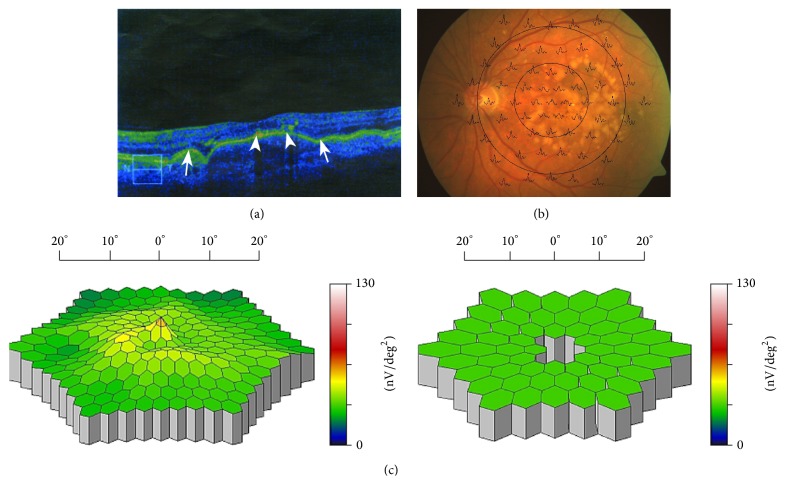
(a) SD-OCT of the left eye of a patient with the dry form of AMD before initiation of rheohemapheresis treatment. A large photoreceptor IS/OS junction defect marked by arrows at its beginning and end. Only the top of the surface DPED under the central foveola shows remnants of previous photoreceptor IS/OS junction detachment (indicated by arrowheads) in the form of degraded degenerated material of the original junction. The photoreceptor IS/OS junction is normally attached to the RPE only peripherally from the arrows. Visual acuity at this stage was 20/80 (0.25). (b) Multifocal electroretinography. Superposition of mfERG responses to the fundus of the left eye of the patient from (a). (c) A three-dimensional image of the electrical activity of the retina. Left: A three-dimensional image of the electrical activity of the retina of the left eye of the patient from (a) and (b) compared to the normal-for-age image on the right (decrease of foveal and parafoveal responses below the normal range, i.e., grey-colored central depressions bordered by green, i.e., within normal range responses).

**Figure 3:**
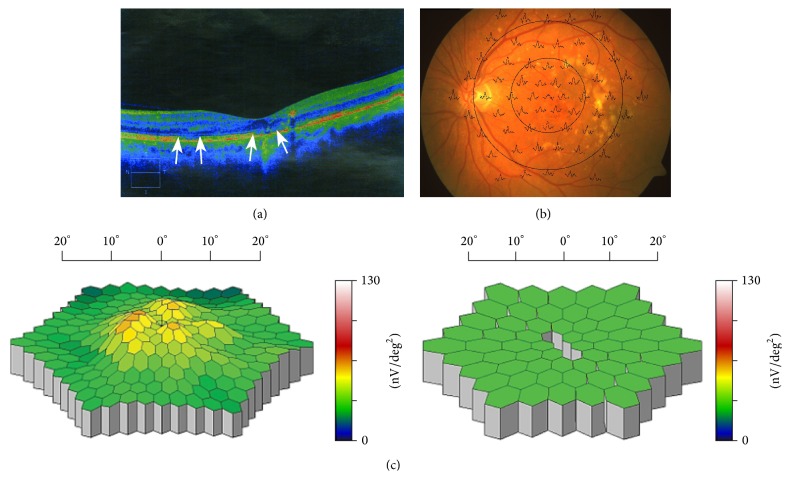
(a) SD-OCT of the same left eye shown in Figure 2(a), taken 2.5 years after rheohemapheresis treatment. Dry form of AMD. In addition to the perfect attachment of the original DPED, there is evident attachment and restoration of the previously detached and degeneratively damaged photoreceptor IS/OS junction, with the exception of two small defects in the central fovea and to the left of it (these defects are located between both the left and right pair of arrows). To the right of the central defect, there is also a small portion of the IS/OS junction which is not visible because it becomes a part of the vertically oriented optical shadow under a dense particle. The remainder of the original junction degeneration is offset towards the inner part of the retina into its plexiform layer. The abovementioned vertical optical shadow does not preclude the presence of the junction section already attached to its intact neighboring sections. Clear morphologic rectification of the position and structure of the photoreceptor IS/OS junction corresponds to the restoration of visual acuity of this eye (BCVA 20/25 (0.8) and improvement of mfERG). (b) Multifocal electroretinogram—responses after RHF. Superposition of mfERG responses to the fundus of the left eye of the patient from (a). (c) A three-dimensional image of the electrical activity of the retina after RHF. Left: A three-dimensional image of the electrical activity of the retina of the left eye of the patient from (a) with increased parafoveal activity, compared to the normal-for-age image on the right (increase of parafoveal activity moves back into the normal range, illustrated by the change from grey depressions to green columns in the parafoveal region).

In general, retinal activity remained stable or even improved in treated patients with early decrease or complete disappearance of DPED and detachment of the photoreceptor IS/OS junction, along with preservation of its integrity or development of only small defects in the parafoveal region (Figures 2(a), 2(b), 2(c), 3(b), and 3(c)). In contrast, in patients with long-lasting or persistent DPED with detachment of the IS/OS junction and development of its defects or even development of CNV, retinal activity was even reduced.

## 4. Discussion

The photoreceptor IS/OS junction layer is currently receiving attention from many researchers [10, 18–22]. According to Baba et al. [19], the state of this thin hyper-reflective layer, which lies above the RPE layer, directly correlates with BCVA after successful macular hole repair. BCVA deterioration has proved to be an early indicator of transformation to the wet form of AMD [1]. We have verified the direct correlation of the state of the IS/OS photoreceptor junction and BCVA on patients with Stargardt disease [18]. Disruption of the IS/OS junction is associated with poor vision in uveitic macular edema and retinitis pigmentosa [22, 23].

As a result of our findings, we can add the IS/OS junction as another indicator of the risk of transformation to the wet form of AMD. The detachment of the photoreceptor IS/OS junction (Figure 1) is usually located at the top of the area affected by drusenoid retinal pigment epithelium detachment (DPED). Sikorski et al. found that rupture of IS/OS photoreceptor junction detachment is directly associated with the emergence of submacular neovascularization [10]. Schuman et al. reported thinning of the IS/OS photoreceptor layer and the progress of its changes over drusen and DPED [20].

In the present study, we found reattachment of the photoreceptor IS/OS junction layer in the study group. After the 2.5-year follow-up, 8 eyes were reattached without IS/OS junction defect after RHF; the IS/OS junction defect was partially reattached in another 8 of the treated eyes. However, in only 4 of these eyes (18.1 % of the entire sample), the defect reached the central fovea. The fact that, after rheohemapheresis, patients only rarely developed the photoreceptor IS/OS junction defect affecting the central foveola and that mean visual acuity actually slightly improved in the majority of these patients is considered an obvious interdependence.

In control patients, we did not observe full restoration (reattachment) of a photoreceptor IS/OS junction layer when detached at the baseline. Drusenoid retinal pigment epithelium detachment usually progresses (which was observed in 7 eyes); otherwise, rupture occurs—caused by submacular neovascularization development, as has also been observed by other authors [24, 25]. The photoreceptor IS/OS junction layer rupture is usually apparent in patients with neovascularization. The remaining fluid as well as the fluid under the detached drusenoid retinal pigment epithelium then spreads into the inner retinal layers and can cause macular edema.

In the control group, we observed 6 cases (33.3%) of submacular choroidal neovascularization. The following is one possible mechanism of its development: the existing DPED was later accompanied by a detached layer adjoined to it from above (i.e., by detachment of the photoreceptor IS/OS junction). DPED and IS/OS layer detachment gradually grew in size and eventually ruptured due to the development of submacular choroidal neovascularization. As evidence of progression to the wet form of AMD, fluid inside and underneath the inner retinal layer appeared on SD-OCT. These findings are supported by the findings of occult submacular choroidal neovascularization using fluorescein angiography.

Patients with preservation of photoreceptor IS/OS junction integrity or development of only small, paracentral defects by early decrease or complete disappearance of the DPED area also exhibited slightly increased retinal activity, which led to significantly higher parafoveal activity in treated patients using mfERG (see Section 3.4). We found a significantly higher amplitude of parafoveal responses in eccentricity between 1.8° and 7° in treated patients 2.5 years after initiation of the treatment (*P* = 0.04). In contrast, in patients with development of IS/OS junction defects or even progression of the disease to its wet form, central retinal activity and visual acuity were even reduced. Development of photoreceptor IS/OS junction defects in the control group probably contributed to the slight decrease of electrical retinal activity in the parafoveal and paramacular regions of eccentricity between 1.8° and 13° using mfERG. This may be due to the long-term accumulation of fluid between the RPE and IS/OS junctions, which causes “stretching” of photoreceptor outer segments, thus leading to their malfunction even without the development of associated RPE atrophy [10]. Insignificant changes of foveal activity could result from the greater vulnerability of foveal cones.

Our study has some limitations. The question of treating the dry form of AMD by rheohemapheresis has not yet been fully answered. Case series, two controlled trials and five completed randomized controlled trials, have reported the efficacy of rheohemapheresis in treating dry AMD [6]. These studies (inclusive of our two [3, 26]) have shown an improvement in the number of lines that can be read on ETDRS charts, improvement in the Pepper Visual Skills for reading tests, a decrease in viscosity parameters, shortening of arteriovenous passage times, and improvement in electroretinograms. The studies have shown improvements shortly after completion of treatment, lasting up to four years [6, 18]. Considerable confusion has been aroused by the preliminary results from the Mira 1 study—an extensive, sham-controlled, randomized, multicenter trial conducted in the USA. Its encouraging first results were later challenged and a clear positive effect was not proved [27, 28]. However, analysis revealed that 37% of treated patients and 29% of control patients were protocol violators who did not fulfill the trial's inclusion criteria of AMD leading to bias in the study's final outcome. Excluding those subjects who had vision loss due to other causes, this trial demonstrated significant improvement with treatment but the trial was underpowered by US FDA licensure. The largest controlled trial to date was conducted by the RheoNet registry. Two hundred and seventy-nine patients with dry AMD were treated and compared to 55 untreated controls. In the treated group, visual acuity gain greater than or equal to one ETDRS line was seen in 42% compared to an improvement in 26% of controls. Vision loss greater than or equal to one ETDRS line was seen in 17% of the treated patients versus 40% of controls. These were statistically significant differences. The ASFA (American Society for Apheresis) issues directives (guidelines) for individual procedures in various diseases; according to the latest criteria from 2013, rheohemapheresis is newly classified as a category IB treatment (first line therapy) [6].

The second limitation of this study is obviously the small number of patients, despite the relatively long follow-up. The clinical impression of the importance of the IS/OS layer was established at the beginning of our research 7 years ago, but only patients with follow-up times longer than 2.5 years, that is, in the case of 40 eyes (22 eyes in the study group and 18 control eyes), were randomized for assessment of morphological and functional changes to the IS/OS layer. Our results will need to be confirmed on a larger number of patients in the future.

## 5. Conclusion

With the use of rheohemapheresis, preservation of photoreceptor IS/OS junction layer coherence in the fovea of patients with high-risk dry AMD can be achieved, even when drusenoid retinal pigment epithelium detachment is already present. After rheohemapheresis, there was a significant morphological improvement of the damaged IS/OS layer, as evidenced by reduction of the scope of the defect or reattachment of the detached IS/OS layer either with or even without the defect of this layer. After 2.5 years of follow-up, better visual acuity, reduced DPED size, and improvement of some functional parameters (electroretinography findings) were observed in the study group.
